# The Effects of the Great East Japan Earthquake on Perinatal Outcomes: Results of the Pregnancy and Birth Survey in the Fukushima Health Management Survey

**DOI:** 10.2188/jea.JE20210444

**Published:** 2022-12-05

**Authors:** Hyo Kyozuka, Tsuyoshi Murata, Shun Yasuda, Kayoko Ishii, Keiya Fujimori, Aya Goto, Seiji Yasumura, Misao Ota, Kenichi Hata, Kohta Suzuki, Akihito Nakai, Tetsuya Ohira, Hitoshi Ohto, Kenji Kamiya

**Affiliations:** 1 Department of Obstetrics and Gynecology, Fukushima Medical University School of Medicine, Fukushima, Japan; 2 Radiation Medical Science Center for the Fukushima Health Management Survey, Fukushima Medical University, Fukushima, Japan; 3 Center for Integrated Science and Humanities, Fukushima Medical University, Fukushima, Japan; 4 Department of Public Health, Fukushima Medical University School of Medicine, Fukushima, Japan; 5 Department of Midwifery and Maternal Nursing, Fukushima Medical University School of Nursing, Fukushima, Japan; 6 Fukushima Society of Obstetrics and Gynecology, Fukushima, Japan; 7 Department of Health and Psychosocial Medicine, Aichi Medical University School of Medicine, Aichi, Japan; 8 Nippon Medical School Tamanagayama Hospital, Tokyo, Japan; 9 Department of Epidemiology, Fukushima Medical University School of Medicine, Fukushima, Japan

**Keywords:** earthquake, nuclear accident, preterm delivery, low-birth-weight infant, congenital anomaly

## Abstract

There are limited studies on the long-term effects of natural/environmental disasters, especially nuclear disasters, on obstetric outcomes. This study aimed to review the results of perinatal outcomes immediately after the Great East Japan Earthquake (GEJE) and the Fukushima Daiichi Nuclear Power Plant accident, as well as their long-term trends over 8 years, in the Fukushima Health Management Survey. The annual population-based Pregnancy and Birth Survey is conducted as part of the Fukushima Health Management Survey. The Fukushima Prefecture government launched it to assess the health conditions of pregnant women and their neonates after the GEJE. The self-reported questionnaire was sent to 115,976 pregnant women by mail from January 2012, with 58,344 women responding to the questionnaire (50.3% response rate). Pregnancy complications, such as gestational hypertension, respiratory diseases, and mental disorders, increased in some women who were pregnant at the time of the earthquake and immediately after the earthquake. However, the direct effects on newborns, such as preterm birth, low birth weight, and congenital anomalies, were not immediately clear after the earthquake. Although there were significant differences in the occurrence of preterm birth and low birth weight among the districts, there was no change in the occurrences of preterm birth, low birth weight, or anomalies in newborns in Fukushima Prefecture from the fiscal year 2011 to the fiscal year 2018. Therefore, the long-term effects of the post-disaster radiation accident on perinatal outcomes are considered to be very small.

## INTRODUCTION

**Figure 1.**
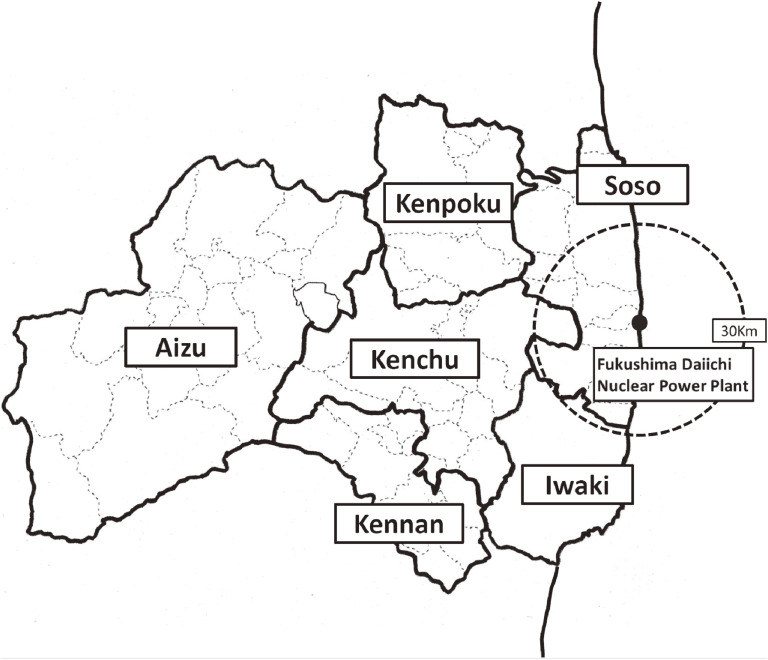
Geographic information of the Fukushima Prefecture. Geographic districts are classified into seven areas (Kenpoku, Kenchu, Kennan, Soso, Iwaki, Aizu, and Minami-Aizu). The Aizu and Minami-Aizu areas were combined and called the Aizu area.

The most devastating event in recent Japanese history is the Great East Japan Earthquake (GEJE), which occurred on Mar 11, 2011, along with the subsequent tsunami and nuclear accident at the Fukushima Daiichi Nuclear Power Plant. In the Fukushima Prefecture, thousands of deaths occurred due to the tsunami. The devastation affected many people living in the coastal areas of the Soso and Iwaki districts. After the power plant accidents, many people (including pregnant women) living in coastal areas were suddenly forced to evacuate by government order. The Aizu district, located in a mountainous region in Fukushima Prefecture far from the power plant, was hardly affected by the nuclear accident compared to the coastal regions (Figure 1). After the disaster, Fukushima Prefecture launched the Fukushima Health Management Survey (FHMS).^1^^,^^2^ This population-based study includes geographical and birth information to evaluate pregnancy outcomes and provide valuable data on the health effects of low radiation doses and disaster-related stress in 2011. Although several studies reported the association between disaster and perinatal health, only a few studies examined perinatal health in disasters, such as Fukushima, where the earthquake, tsunami, and radiation exposure occurred in combination.^3^ Few studies reported chronological trends in perinatal outcomes after the disaster. In particular, due to the concerns about radiation exposure in Fukushima, the chronological trends in pregnancy outcomes after the GEJE are of worldwide interest; the FHMS has maintained data from the investigation on the effects of this disaster on pregnancy and infant care.

This study examined the status of perinatal health in Fukushima Prefecture before and after the GEJE and their time-series trends over 8 years by reviewing articles and reports on perinatal health surveyed by the FHMS. Based on these results, we discuss the impact of the disasters, including radiation exposure, on perinatal health in Fukushima.

## METHODS

### Survey design

Fukushima Medical University launched the Pregnancy and Birth Survey as part of the FHMS in fiscal year (FY) 2011.^1^^,^^2^^,^^4^ The survey included women who received the maternal and child health handbook between August 1 of the previous year and July 31 of the corresponding year. The maternal and child health handbook is a unique perinatal healthcare initiative in Japan. The handbook helps maintain a record of women’s prenatal and postnatal checkups by physicians.

### Target population

The self-reported questionnaire was sent to 115,976 pregnant women by mail between January 2012 and December 2019 (FY2011, 16,001; FY2012, 14,516; FY2013, 15,218; FY2014, 15,125; FY2015, 14,572; FY2016, 14,154; FY2017, 13,552; FY2018, 12,838). The mothers were asked to complete the questionnaire while referring to their maternal and child health handbooks described above. In total, 58,344 women (50.3% response rate) responded to the questionnaire. The number of cases from 2011 to 2018 was 9,316, 7,181, 7,260, 7,132, 7,031, 7,326, 6,449, and 6,649, respectively.^5^

### Maternal information and obstetrics outcome

The self-reported questionnaire included maternal information, such as the geographic district where the pregnant women received their maternal and child health handbook, delivery date, maternal age at delivery, single or multiple gestational pregnancies, gestational weeks at delivery, mode of pregnancy, and mode of delivery. It also included neonatal information, such as neonatal birth weight, sex of the newborn, and the presence of anomalies in the newborn. There were six classifications for the geographic district: Kenpoku, Kenchu, Kennan, Soso, Iwaki, and Aizu (Figure 1). Soso and Iwaki are on the coastal areas of Fukushima Prefecture. Soso is nearest the nuclear power plant, and many of its residents were forced to evacuate after the nuclear accident. Kenpoku, Kenchu, and Kennan are in the center of Fukushima Prefecture. Kenpoku reportedly has relatively higher radiation levels than Kenchu and Kennan.^6^^,^^7^ The Aizu region is mountainous and far from the nuclear power plant, and radiation levels are the lowest in the Prefecture. Deliveries before 37 gestational weeks were defined as preterm birth, and birth weights less than 2,500 g were defined as low birth weight.^8^ The mode of pregnancy was categorized as natural pregnancy or fertility treatment, such as ovulation induction, artificial insemination, and in vitro fertilization. The mode of delivery was categorized as vaginal delivery or caesarian section. The following major anomalies were reported in the newborns: cataracts, cardiac malformation, kidney, and urinary tract malformation, spina bifida, microcephaly, hydrocephalous, cleft lip and palate, intestinal atresia (esophagus, duodenum, ileum), imperforate anus, polydactylism, and syndactylism. Every anomaly reported on the questionnaire was defined as a major anomaly.

### Ethical considerations

The local ethics review committee of the author’s institution approved this study (Approval No. 1317), and all participants provided written informed consent.

## RESULTS

### The incidence of pregnancy complications among pregnant women during the disaster

We report pregnancy complications among pregnant women during the disaster using the results of the FHMS, which targeted women who gave birth during 2011–2012. Pregnant women were divided into four groups according to pregnancy trimester during the disaster (first, second, third trimester, or conception after the disaster). As a result, the third trimester of pregnancy at the time of the disaster was associated with hypertensive pregnancy disorder for the women living in the areas most exposed to the disaster.^9^

**Figure 2.**
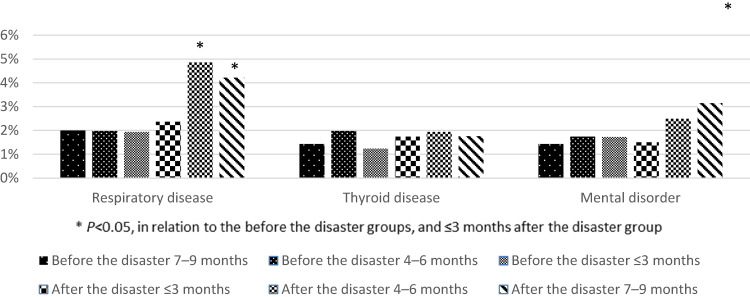
Proportions of medical complications among the pregnant women before and after the disaster. ^*^P < 0.05, in relation to the ≤3 months before the disaster group.

Data from 12,300 women who became pregnant in Fukushima Prefecture in the 9 months before and after the disaster were collected and analyzed.^10^ The results showed no obstetric-related adverse events among the women who became pregnant in the 9 months before the disaster.^10^ In contrast, there was an increased incidence of medical complications, such as respiratory diseases and mental disorders, among the women who became pregnant within 6 months of the earthquake (Figure 2).^10^

Suzuki et al examined the association between changes in medical institutions for perinatal care and gestational duration after the GEJE in 5,593 survey participants. They experienced the earthquake between the fourth and 37th weeks of their gestational period.^11^ The results showed that pregnant women who changed perinatal checkup institutions due to medical indications had a significantly shorter gestation duration and more preterm births than those who visited only one institution. However, self-referral, which could indicate post-disaster relocation, was not significantly associated with shorter gestation and preterm birth,^11^ suggesting that the GEJE and the subsequent accident at the Fukushima Daiichi Nuclear Power Plant did not have a direct impact on gestational duration.

### The incidence of stillbirth, preterm birth, low birth weight, and congenital anomalies during the disaster

**Figure 3.**
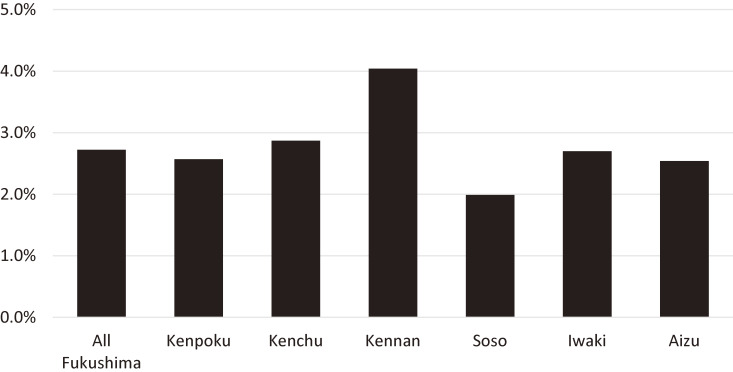
Incidence rate of congenital anomalies by regional areas

In Fukushima Prefecture during the first year after the disaster, the incidence rates for stillbirth (over 22 completed gestational weeks), preterm birth, low birth weight, and congenital anomalies were 0.25%, 4.4%, 8.7%, and 2.72%, respectively.^4^ These rates are similar to rates recently reported elsewhere in Japan. There were no significant differences in the incidence rate of stillbirth or preterm birth among the regional areas, while the incidence rate of low birth weight varied; that of Iwaki was the highest (10.6%) and Kenpoku was the lowest (7.6%).^4^ There were no significant differences in the incidence rate of all congenital anomalies among the regional area (*P* for difference = 0.275), although the rate of Kennan was somewhat higher than other regional areas (Figure 3).^4^

**Figure 4.**
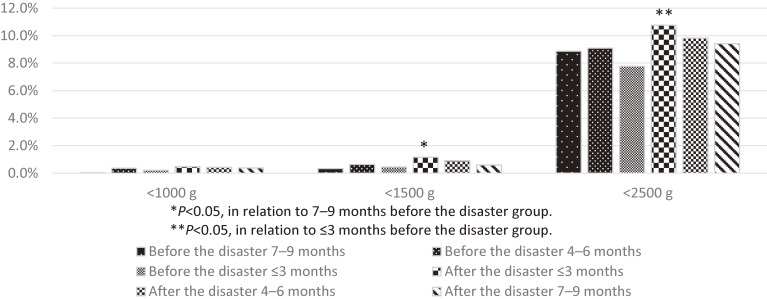
Incidence rate of low birth weight in the pregnant women before and after the disaster. ^*^P < 0.05, in relation to 9–7 months before the disaster group. ^**^P < 0.05, in relation to ≤3 months before the disaster group.

**Figure 5.**
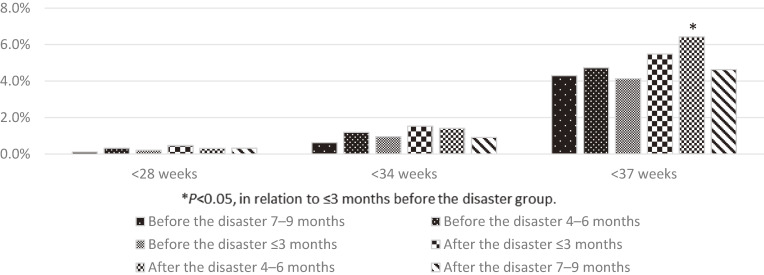
Incidence rate of preterm birth among the pregnant women before and after the disaster. ^*^P < 0.05, in relation to ≤3 months before the disaster group.

The analysis of the same 12,300 participants as above showed an increased incidence of low birth weight (<1,500 g and <2,500 g) and preterm birth among women who became pregnant within 6 months of the earthquake (Figure 4 and Figure 5).^10^ We also evaluated the incidence and obstetric outcomes of women who became pregnant using assisted reproductive technology procedures in Fukushima Prefecture and reported that the impact of the disaster was minimal. The proportion of women who became pregnant using in vitro fertilization and embryo transfer decreased during the first 2 months after the earthquake. It returned to pre-disaster levels in the third month after the earthquake. For women who became pregnant without in vitro fertilization and embryo transfer, the incidence of preterm birth and low birth weight increased after the earthquake.^12^

The incidence rate for small for gestational age (SGA) status among newborns delivered by women who were pregnant at the time of the disaster in Fukushima Prefecture was 5.6% (325 of 5,790).^13^ The regional proximity to the disaster and gestational age did not affect the incidence of SGA. In the multivariate analysis, gestational hypertension (also known as pregnancy-induced hypertension) was higher in the SGA group and was an independent risk factor for SGA.^13^

### Trends in preterm birth, low birth weight, and congenital anomalies from FY2011 to FY2018

**Figure 6.**
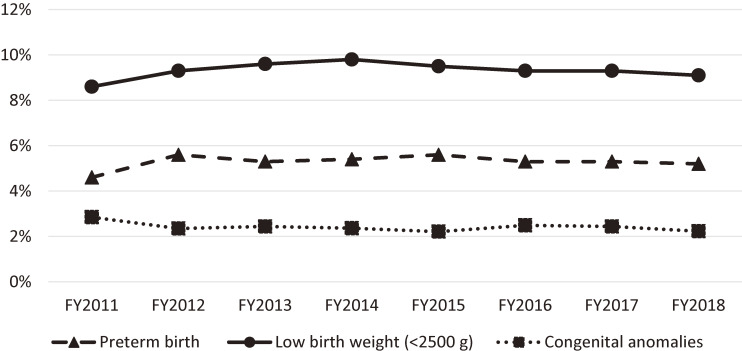
Trends in incidence rate of preterm birth, low birth weight, and congenital anomalies from FY 2011 to FY 2018

Figure 6 shows the trends in preterm birth, low birth weight, and congenital anomalies from FY2011 to FY2018.^5^ There was no change in incidence rate of preterm birth, low birth weight, and congenital anomalies during the survey period. The incidence of preterm births and low birth weight ranged from 4.8% to 5.8% and from 8.9% to 10.1%, respectively, during the survey period. Meanwhile, 5.7% and 9.4% of births nationwide were reported as preterm births and low birth weight, respectively, according to the 2017 Vital Statistics, and the survey results in Fukushima were similar to the national results. The incidence of congenital anomalies ranged from 2.19% to 2.85% during the survey period. Since the general incidence of congenital anomalies is reported to be 3% to 5%, the incidence in Fukushima was considered to be equal to or lower than these values.

## DISCUSSION

The Pregnancy and Birth Survey of the Fukushima Health Management Survey is the first population-based survey to examine the long-term trends of perinatal outcomes among pregnant women in Fukushima Prefecture after the disaster. This survey revealed that pregnancy complications, such as gestational hypertension, respiratory diseases, and mental disorders, increased in some women who were pregnant at the time of the earthquake and immediately after the earthquake. However, the direct effects on newborns, such as preterm birth, low birth weight, and congenital anomalies, were unclear, either immediately after the earthquake or for several years after the earthquake. Furthermore, there is no association between radiation exposure dose and perinatal outcomes.^14^ Therefore, we can conclude that there is no evidence of a direct effect of radiation exposure on perinatal outcomes due to the radiation accident of Fukushima Prefecture.

Disasters potentially influence a range of reproductive outcomes.^15^ Numerous studies examined the effects of exposure to disasters on pregnancy outcomes, such as the World Trade Center Disaster, the bombing attacks in Serbia, and the Madrid train bombing; environmental and chemical disasters, such as the Bhopal gas release in India, the Three Mile Island accident, and the Chernobyl accident; and natural disasters, such as earthquakes, hurricanes, floods, and ice streams.^3^ The GEJE and Fukushima Daiichi nuclear accidents formed a complex disaster because they included the natural disaster of the Great Earthquake and tsunami and the environmental/technical disaster of the nuclear power plant accident. However, the Japan Environment and Children’s Study conducted in Fukushima Prefecture examined perinatal outcomes using the results of 12,804 births in the prefecture between 2011 and 2014.^16^ The prevalence of preterm birth, low birth weight, and neonatal anomalies were 5.6%, 9.5%, and 1.7%, respectively,^16^ which were not so different from the results of our study.

### Fukushima disaster and preterm birth and low birth weight

There are conflicting findings on the association between environmental/chemical disasters and gestational age and birth weight. Goldman et al reported that the Love Canal disaster in the United States showed no significant association with gestational age among 227 residents.^17^ However, Levi et al reported that the Chernobyl accident affected gestational age and maternal anxiety among 88 Swedish women early in their pregnancy at the time of the disaster.^18^ In contrast to our study, an earthquake disaster in China increased the risk of preterm birth. Tan et al compared the incidence of preterm birth between 6,638 pregnant women before the disaster and 6,365 pregnant women after.^19^ The incidence of preterm birth was 5.6% and 7.4%, respectively, which was significantly higher after the disaster (*P* < 0.01). In Japan, the incidence of high-risk pregnancies has increased due to advanced maternal age and complicated pregnancies^20^^,^^21^; the post-disaster incidence rates of preterm birth <37 gestational weeks (5.7%) and low birth weight <2,500 g (9.4%)^22^ have been almost stable.

Several studies have reported that environmental and natural disasters increase the risk of low birth weight.^23^^–^^25^ However, several studies have reported no effect of these disasters on birth weight.^17^^,^^26^ With regard to the GEJE and Fukushima Daiichi nuclear accident, we reported that there was no evidence that the disaster increased the incidence of SGA births in Fukushima Prefecture during the 8 years after the disaster. Using an institutional investigation in a coastal area where the maximum catastrophic damage had occurred in the GEJE,^27^ Leppold et al reported no marked increase in the proportion of preterm birth and low birth weight in any year after the disaster (merged post-disaster risk ratio of preterm birth: 0.68; 95% confidence interval [CI], 0.38–1.21 and low birthweight: 0.98; 95% CI, 0.64–1.51).^27^ In Japan, pregnant women could have better access to relief programs or might receive adequate support from their family, society, and the government during disasters.^28^ Although the effects on newborns are unclear, it has been reported that the proportion of pregnant women with depressive symptoms increased after the GEJE due to the sudden evacuation of numerous pregnant women and the drastic changes in their living environment.^28^^–^^30^ Therefore, we need to continue examining the direct effects of the earthquake and radiation accident and the indirect effects, such as long-term evacuation.

### Fukushima disaster and congenital anomalies

The association between disasters and congenital anomalies is a major public concern. Several major environmental and technical disasters have been related to congenital anomalies, including the nuclear reactor accidents at Chernobyl in 1986 and Three Mile Island in 1979. The accident at Chernobyl involved a much larger radiation dose exposure and affected more people than the Three Mile Island and Fukushima incidents. Reviews on the effect of the Chernobyl disaster indicated increased microcephaly and neural tube defects.^31^^–^^33^ However, the incidence of most congenital disabilities did not increase in most European countries.^34^^–^^36^ It was widely reported that 2–3% of all newborns have a major congenital abnormality that is detectable at birth.^37^^,^^38^ From 2011 to 2016, the incidence of congenital disabilities in Japan was 2.43–2.59%, according to a report of the International Clearinghouse for Birth Defects Surveillance and Research Japan Center,^39^ although this has not increased since the GEJE. Using the Japanese birth cohort study that included 12,804 pregnant women in Fukushima Prefecture, Kyozuka et al reported that the prevalence of major congenital anomalies at delivery between 2011 and 2014 in the prefecture was 1.6% to 3.2%, depending on maternal age,^16^ and these rates were similar to the national rates.

Nevertheless, a survey of hospital discharge records for cryptorchidism showed an increased discharge rate for cryptorchidism nationwide after the GEJE.^40^ However, Kojima et al reported that it is difficult to clarify cryptorchidism prevalence because of complexities in design settings for epidemiological surveys of this disease. They rejected the hypothesis that cryptorchidism increased in Japan due to the Fukushima Daiichi Nuclear Power Plant accident.^41^ Hirai et al, employing the All Japanese Cardiovascular Surgery Database, revealed no increase in the number of patients with congenital heart disease from 2010 to 2013.^42^

### Strengths and limitations

The Pregnancy and Birth Survey has several strengths. In Japan, few epidemiological studies involve pregnant women in a community. Large-scale studies and data supported by the government are considered valuable. In addition, we obtained relatively accurate data on gestational ages and birth weights from the participants from their maternal and child health handbook. Nevertheless, the survey has potential limitations. First, the response rate was only approximately 50–60% throughout the study period. Therefore, the actual incidence of negative outcomes could have been overestimated if there was an overrepresentation of women affected the most by the disasters, especially those who were pregnant between 2011 and 2012. Second, given that this study used a self-administered questionnaire, we assume that the mothers answered correctly, especially regarding fetal anomalies.

### Conclusion

In conclusion, the results of the Pregnancy and Birth Survey indicate that the effects of GEJE and the subsequent nuclear accident on pregnant women are negligible in the long term. Improving the understanding of the adverse reproductive effects of disasters requires as much preparedness as required for the emergency response to prevent mortality and morbidity. Further studies should examine whether the disaster caused psychological complications or early pregnancy loss, such as miscarriage and abortion. Due to the relatively high incidence of anxiety and potential long-lasting negative effects on the mental and physical health of mothers, their children, and even other family members, the findings of the Pregnancy and Birth Survey could have significant public health implications.

## References

[1] Yasumura S, Hosoya M, Yamashita S, ; Fukushima Health Management Survey Group. Study protocol for the Fukushima Health Management Survey. J Epidemiol. 2012;22(5):375–383. 10.2188/jea.JE20120105 22955043 PMC3798631

[2] Yasumura S, Ohira T, Ishikawa T, . Achievements and current status of the Fukushima Health Management Survey. J Epidemiol. 2022;32(Suppl 12):S3–S10. 10.2188/jea.JE20210390 PMC9703928 36464298

[3] Harville E, Xiong X, Buekens P. Disasters and perinatal health: a systematic review. Obstet Gynecol Surv. 2010;65:713–728. 10.1097/OGX.0b013e31820eddbe 21375788 PMC3472448

[4] Fujimori K, Kyozuka H, Yasuda S, ; Pregnancy and Birth Survey Group of the Fukushima Health Management Survey. Pregnancy and birth survey after the Great East Japan Earthquake and Fukushima Daiichi Nuclear Power Plant accident in Fukushima prefecture. Fukushima J Med Sci. 2014;60(1):75–81. 10.5387/fms.2014-9 25030719

[5] Radiation Medical Science Center for the Fukushima Health Management Survey. Proceedings of the 37th Prefectural Oversight Committee Meeting for Fukushima Health Management Survey (in Japanese). https://www.pref.fukushima.lg.jp/uploaded/attachment/369430.pdf Accessed Sep 17, 2021.

[6] Ishikawa T, Yasumura S, Akahane K, . External doses available for epidemiological studies related to the Fukushima Health Management Survey: first 4-month individual doses and municipality-average doses for the first year. J Epidemiol. 2022;32(Suppl 12):S11–S22. 10.2188/jea.JE20210166 PMC9703927 36464295

[7] Ishikawa T, Yasumura S, Ozasa K, . The Fukushima Health Management Survey: estimation of external doses to residents in Fukushima Prefecture. Sci Rep. 2015;5:12712. 10.1038/srep12712 26239643 PMC4523853

[8] Kyozuka H, Fujimori K, Hosoya M, ; Japan Environment and Children’s Study (JECS) Group. The effect of maternal age at the first childbirth on gestational age and birth weight: the Japan Environment and Children’s Study (JECS). J Epidemiol. 2019;29(5):187–191. 10.2188/jea.JE20170283 30078812 PMC6445800

[9] Kyozuka H, Murata T, Yasuda S, ; Pregnancy and Birth Survey Group of the Fukushima Health Management Survey. The effect of the Great East Japan Earthquake on hypertensive disorders during pregnancy: a study from the Fukushima Health Management Survey. J Matern Fetal Neonatal Med. 2020;33(24):4043–4048. 10.1080/14767058.2019.1594763 30880508

[10] Hayashi M, Fujimori K, Yasumura S, Goto A, Nakai A. Obstetric outcomes in women in Fukushima Prefecture during and after the Great East Japan Earthquake and Fukushima Nuclear Power Plant Accident: the Fukushima Health Management Survey. Open J Obstet Gynecol. 2016;06:705–713. 10.4236/ojog.2016.612088

[11] Suzuki K, Goto A, Fujimori K. Effect of medical institution change on gestational duration after the Great East Japan Earthquake: the Fukushima Health Management Survey. J Obstet Gynaecol Res. 2016;42:1704–1711. 10.1111/jog.13102 27528440

[12] Hayashi M, Fujimori K, Yasumura S, Nakai A; Pregnancy and Birth Survey Group of the Fukushima Health Management Survey. Impact of the Great East Japan Earthquake and Fukushima Nuclear Power Plant accident on assisted reproductive technology in Fukushima Prefecture: the Fukushima Health Management Survey. J Clin Med Res. 2017;9(9):776–781. 10.14740/jocmr3105w 28811855 PMC5544483

[13] Yasuda S, Kyozuka H, Nomura Y, . Influence of the Great East Japan Earthquake and the Fukushima Daiichi nuclear disaster on the birth weight of newborns in Fukushima Prefecture: Fukushima Health Management Survey. J Matern Fetal Neonatal Med. 2017;30:2900–2904. 10.1080/14767058.2016.1245718 27718768

[14] Yasuda S, Okazaki K, Nakano H, . Effects of external radiation exposure on perinatal outcomes in pregnant women after the Fukushima Daiichi Nuclear Power Plant accident: the Fukushima Health Management Survey. J Epidemiol. 2022;32(Suppl 12):S104–S114. 10.2188/jea.JE20210252 PMC9703922 36464294

[15] Cordero JF. The epidemiology of disasters and adverse reproductive outcomes: lessons learned. Environ Health Perspect. 1993;101(Suppl 2):131–136. 10.1289/ehp.93101s2131 8243383 PMC1519923

[16] Kyozuka H, Fujimori K, Hosoya M, . The Japan environment and children’s study (JECS) in Fukushima Prefecture: pregnancy outcome after the great east Japan earthquake. Tohoku J Exp Med. 2018;246:27–33. 10.1620/tjem.246.27 30210086

[17] Goldman LR, Paigen B, Magnant MM, Highland JH. Low birth weight, prematurity and birth defects in children living near the hazardous waste site, love Canal. Hazard Waste Hazard Mater. 1985;2:209–223. 10.1089/hwm.1985.2.209

[18] Levi R, Lundberg U, Hanson U, Frankenhacuser M. Anxiety during pregnancy after the Chernobyl accident as related to obstetric outcome. J Psychosom Obstet Gynaecol. 1989;10:221–230. 10.3109/01674828909016696

[19] Tan CE, Li HJ, Zhang XG, . The impact of the Wenchuan earthquake on birth outcomes. PLoS One. 2009;4:e8200. 10.1371/journal.pone.0008200 19997649 PMC2781160

[20] Kyozuka H, Yamaguchi A, Suzuki D, ; Japan Environment and Children’s Study (JECS) Group. Risk factors for placenta accreta spectrum: findings from the Japan environment and Children’s study. BMC Pregnancy Childbirth. 2019;19(1):447. 10.1186/s12884-019-2608-9 31775687 PMC6882023

[21] Yamaguchi A, Kyozuka H, Fujimori K, ; Japan Environment and Children’s Study Group. Risk of preterm birth, low birthweight and small-for-gestational-age infants in pregnancies with adenomyosis: a cohort study of the Japan Environment and Children’s Study. Acta Obstet Gynecol Scand. 2019;98(3):359–364. 10.1111/aogs.13498 30367455

[22] Mother’s and children’s Health Organization. In: Maternal and Child Health Statistics of Japan. Live Births and Percentages by Period of Gestation; vol 49; 2019:1980–2011.

[23] Chang HL, Chang TC, Lin TY, Kuo SS. Psychiatric morbidity and pregnancy outcome in a disaster area of Taiwan 921 earthquake. Psychiatry Clin Neurosci. 2002;56:139–144. 10.1046/j.1440-1819.2002.00948.x 11952916

[24] Xiong X, Harville EW, Mattison DR, Elkind-Hirsch K, Pridjian G, Buekens P. Exposure to Hurricane Katrina, post-traumatic stress disorder and birth outcomes. Am J Med Sci. 2008;336:111–115. 10.1097/MAJ.0b013e318180f21c 18703903 PMC2635112

[25] Tong VT, Zotti ME, Hsia J. Impact of the Red River catastrophic flood on women giving birth in North Dakota, 1994–2000. Matern Child Health J. 2011;15:281–288. 10.1007/s10995-010-0576-9 20204482

[26] Hamilton BE, Sutton PD, Mathews TJ, Martin JA, Ventura SJ. The effect of Hurricane Katrina: births in the U.S. Gulf Coast region, before and after the storm. Natl Vital Stat Rep. 2009;58(2):1–28, 32. 19754006

[27] Leppold C, Nomura S, Sawano T, . Birth outcomes after the Fukushima Daiichi Nuclear Power Plant Disaster: a long-term retrospective study. Int J Environ Res Public Health. 2017;14:542. 10.3390/ijerph14050542 28534840 PMC5451992

[28] Kyozuka H, Yasuda S, Kawamura M, . Impact of the Great East Japan Earthquake on feeding methods and newborn growth at 1 month postpartum: results from the Fukushima Health Management Survey. Radiat Environ Biophys. 2016;55:139–146. 10.1007/s00411-016-0636-7 26875100 PMC4840221

[29] Goto A, Bromet EJ, Fujimori K; Pregnancy and Birth Survey Group of Fukushima Health Management Survey. Immediate effects of the Fukushima nuclear power plant disaster on depressive symptoms among mothers with infants: a prefectural-wide cross-sectional study from the Fukushima Health Management Survey. BMC Psychiatry. 2015;15:59. 10.1186/s12888-015-0443-8 25885267 PMC4393633

[30] Ishi K, Goto A, Komiya H, . Postpartum mental health of mothers in Fukushima: insights from the Fukushima Health Management Survey (FHMS) 8-year trends. J Epidemiol Revision. (submitted). 10.2188/jea.JE20210385 PMC9703933 36464302

[31] Wertelecki W. Malformations in a Chornobyl-impacted region. Pediatrics. 2010;125:e836–e843. 10.1542/peds.2009-2219 20308207

[32] Wertelecki W, Chambers CD, Yevtushok L, . Chornobyl 30 years later: radiation, pregnancies, and developmental anomalies in Rivne, Ukraine. Eur J Med Genet. 2017;60:2–11. 10.1016/j.ejmg.2016.09.019 27697599

[33] Wertelecki W, Yevtushok L, Kuznietsov I, . Chornobyl, radiation, neural tube defects, and microcephaly. Eur J Med Genet. 2018;61:556–563. 10.1016/j.ejmg.2018.06.005 29908351

[34] Dolk H, Nichols R. Evaluation of the impact of Chernobyl on the prevalence of congenital anomalies in 16 regions of Europe. EUROCAT Working Group. Int J Epidemiol. 1999;28:941–948. 10.1093/ije/28.5.941 10597995

[35] Hoffmann W. Fallout from the Chernobyl nuclear disaster and congenital malformations in Europe. Arch Environ Health. 2001;56:478–484. 10.1080/00039890109602895 11958546

[36] Little J. The Chernobyl accident, congenital anomalies and other reproductive outcomes. Paediatr Perinat Epidemiol. 1993;7:121–151. 10.1111/j.1365-3016.1993.tb00388.x 8516187

[37] Cragan JD, Gilboa SM. Including prenatal diagnoses in birth defects monitoring: experience of the Metropolitan Atlanta Congenital Defects Program. Birth Defects Res A Clin Mol Teratol. 2009;85:20–29. 10.1002/bdra.20508 19089857

[38] Dolk H, Loane M, Garne E. The prevalence of congenital anomalies in Europe. In: Adv Exp Med Biol Posada de la Paz M, Groft S, eds. *Rare Diseases Epidemiology*. Dordrecht: Springer; 2010;686:349–364. 10.1007/978-90-481-9485-8_20 20824455

[39] International clearinghouse for birth defects surveillance and Research, Japan center. https://icbdsr-j.jp/data.html, in Japanese, Accessed September 17, 2021.

[40] Murase K, Murase J, Machidori K, Mizuno K, Hayashi Y, Kohri K. Nationwide increase in cryptorchidism after the Fukushima nuclear accident. Urology. 2018;118:65–70. 10.1016/j.urology.2018.04.033 29751027

[41] Kojima Y, Yokoya S, Kurita N, . Cryptorchidism after the Fukushima Daiichi Nuclear Power Plant accident: causation or coincidence? Fukushima J Med Sci. 2019;65:76–98. 10.5387/fms.2019-22 31915325 PMC7012587

[42] Hirata Y, Shimizu H, Kumamaru H, . Congenital heart disease after the Fukushima Nuclear Accident: the Japan cardiovascular Surgery Database Study. J Am Heart Assoc. 2020;9:e014787. 10.1161/JAHA.119.014787 32613886 PMC7670522

